# Supplementary material for: Optical linewidth of soliton microcombs
Source: Nat Commun. 2022 Jun 7;13:3161. doi: 10.1038/s41467-022-30726-5 (PMC9174472; doi:10.1038/s41467-022-30726-5)
Supplement: Supplementary file 1 — Supplementary Information [file 41467_2022_30726_MOESM1_ESM.pdf]

Optical linewidth of soliton microcombs: Supplementary Information

Fuchuan Lei, Zhichao Ye, Óskar B. Helgason, Attila Fülöp,
Marcello Girardi and Victor Torres-Company

May 6, 2022

Supplementary Note 1: Influence of the dispersive-wave recoil on the location of the fixed point

In the main text, we have discussed the transduction of the pump frequency noise into repetition rate noise induced by the Raman self-frequency shift. In this section, we study the influence of dispersive-wave recoil. The dispersive wave could be introduced by third-order or higher-order dispersion of the microresonator or the mode-coupling induced mode shift [1-3]. First, we analyse the influence of the third-order dispersion (TOD) numerically. As shown in Supplementary Fig. 1a, the location of the fixed point can be changed slightly with the TOD, with all the other parameters being equal. More interestingly, in absence of Raman, TOD can lead to linewidth narrowing as well, and the fixed point could be located either on the blue or red side depending on the sign of β_3. However, the TOD is typically so weak that the fixed point appears outside of the comb spectrum, as shown in Supplementary Fig. 1b.

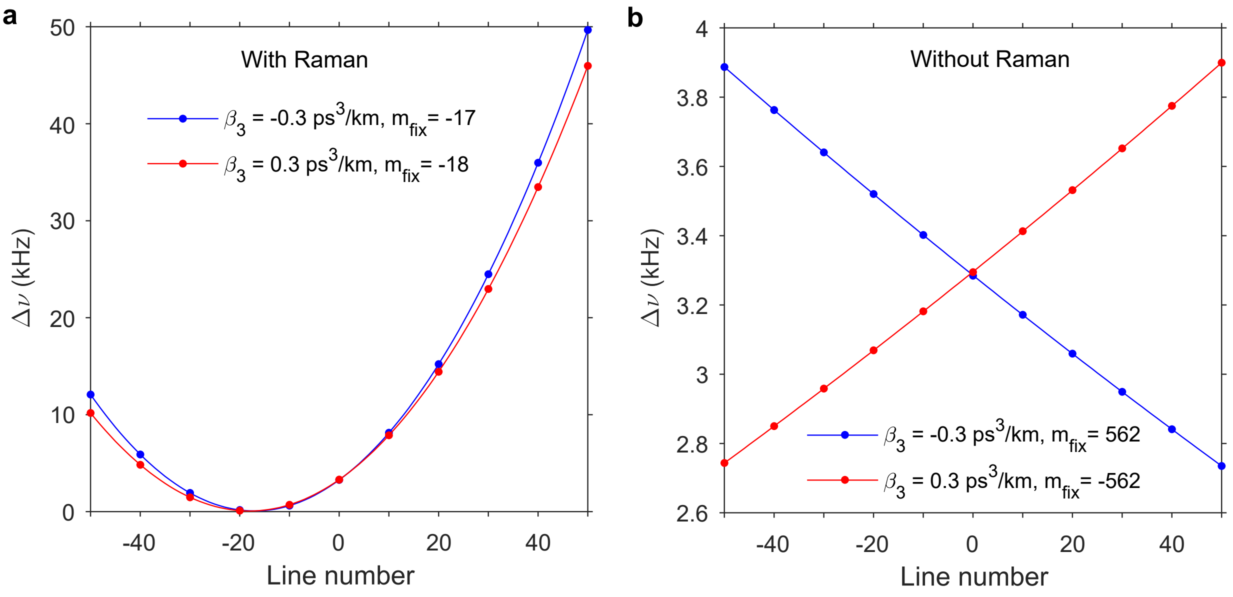

Supplementary Figure 1: The linewith of comb lines induced by pump phase noise in presence of third-order dispersion (TOD). The TOD can modify the linewidth of comb lines, when the Raman is included **a** or excluded **b**.

It has been demonstrated that the mode interaction induced dispersive-wave recoil could balance the Raman self-frequency shift, leading to quiet point, where the repetition rate is insensitive to variation of the pump-resonance detuning [4]. However, the mode interaction is often uncontrollable in single ring cavities. Here, we consider a microcomb generated in a photonic molecule configuration [5], see Supplementary Fig. 2a. The width and height of the two microrings are 1600 nm and 600 nm respectively, resulting in a group velocity

dispersion coefficient of β_2 =100 ps^2/km for mode TE_{00}. The FSRs for the two rings are 100.79 GHz and 99.83 GHz respectively. The two rings feature similar intrinsic Q (5 million) and extrinsic Q (6.5 million) factors. The gap between the two rings is 500 nm, which results in mode coupling between two rings. The coupling induced mode splitting is $2\pi\times 607$ MHz. To generate the soliton, we pump a supermode at 1569.44 nm with on-chip power around 40 mW, with similar operation to [5]. In addition to the pump mode, many other pairs of longitudinal modes from the two microrings are also coupled, leading to a complicated dispersion profile and frequency spectrum, see Supplementary Fig. 2b.

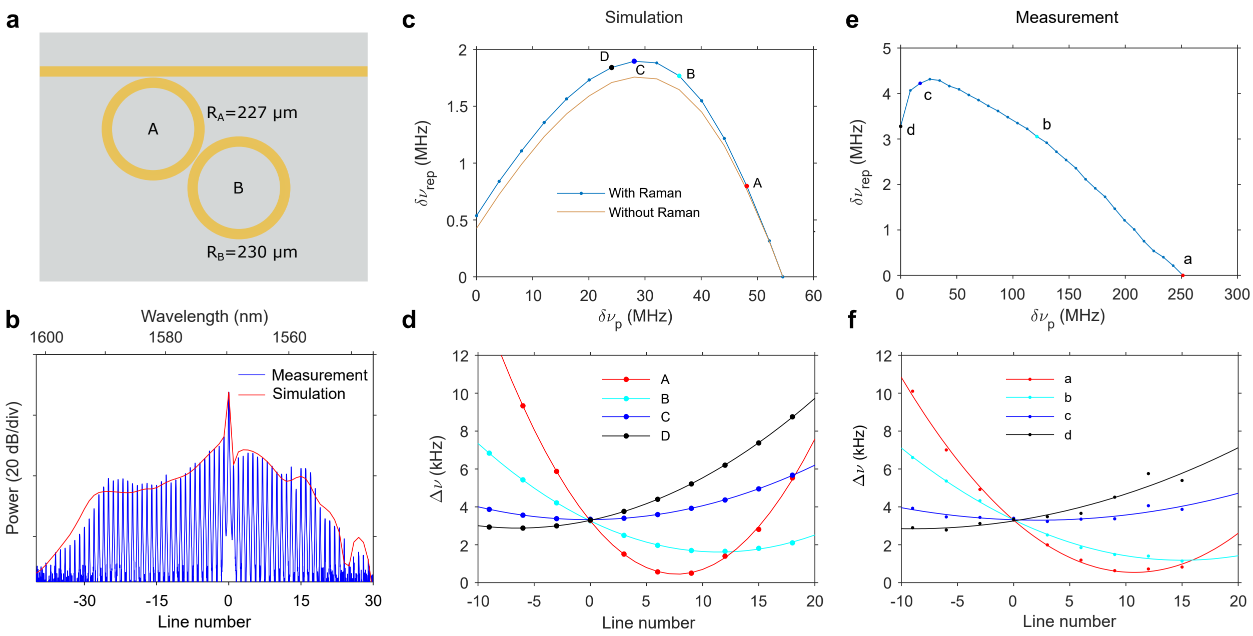

Figure 2: Lorentzian linewidth of photonic molecule soliton microcombs. **a** Schematic of photonic molecule microresonators. **b** Optical spectrum of the photonic molecule microcomb. **c** Simulated dependence of soliton microcomb repetition rate change on the relative pump frequency. **d** Simulated Lorentzian linewidth of comb lines at different pump frequencies. **e-f** are measured results corresponding to **c** and **d**, respectively.

In contrast to the single-cavity microcomb in the main text, the repetition rate does not follow a linear but a parabolic dependence of the pump frequency, see Supplementary Fig. 2c. It means there exists a quiet point in the system. Note that this quiet point is mainly induced by the balance of dispersive waves, as the Raman plays a quite weak effect here (with the Raman term switched off in the simulation, we can still obtain this quiet point, see Supplementary Fig. 2c). The details of the underlying physics deserve further exploration. Here we focus on its impact on the Lorentzian linewidth of comb lines. At the quiet point, there is no significant linewidth narrowing effect as the linewidth of comb lines turns out to be slightly higher than that of the pump due to the shot noise and pump intensity noise. As shown in Supplementary Fig. 2d, we observe the linewidth of comb lines is also a function of the detuning, and the number of the comb line with the minimum linewidth can be modified to be at either the blue or the red side of pump.

These simulated phenomena are also qualitatively confirmed in the experiment, as seen in Supplementary Fig. 2e and 2f. The difference between the simulations and experiments can be attributed to the thermal effect which is not included in the simulations. Unlike the generation of soliton in a single microresonator with anomalous dispersion where the pump is red detuned from the cavity resonance, here the pump is at the blue detuned side of the cavity resonance [5]. Therefore, the system's temperature strongly relies on the pump frequency. The change of temperature would induce the two-cavity detuning and repetition rate change, and even modify the soliton existence condition.

The above results indicate that the elastic tape model appears to apply for a broad range of coherent microcombs, in spite of different physical mechanisms coupling with repetition rate with detuning.

Supplementary Note 2: Influence of the pump linewidth

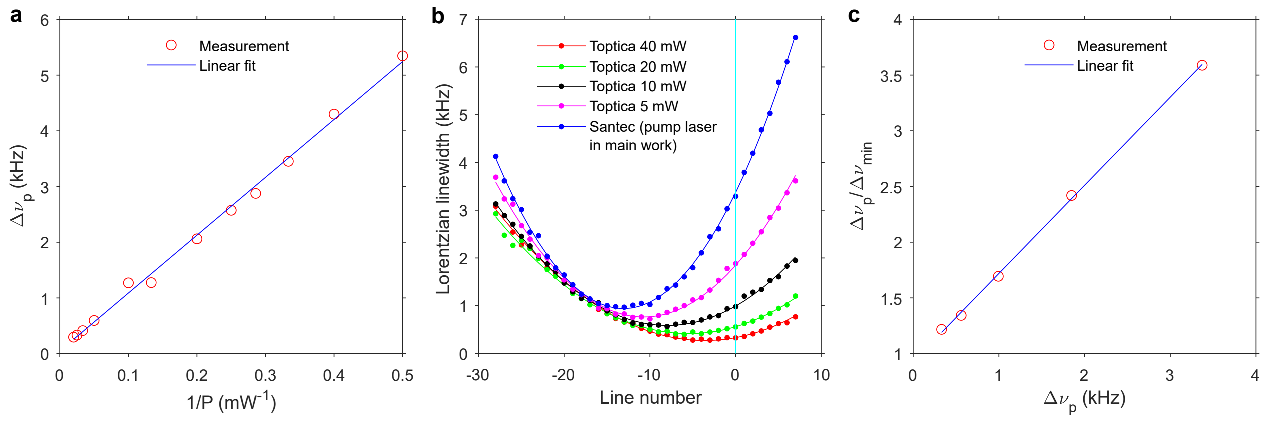

Supplementary Figure 3: Operation of soliton microcomb with a pump with different Lorentzian linewidth. **a** The measured Lorentzian linewidth of a pump laser (Toptica, CTL 1550) at different output powers. **b** The measured Lorentzian linewidth of the soliton microcomb with different pump sources. **c** Relative reduction of Lorentzian linewidth as a function of the pump Lorentzian linewidth.

According to equation (9) in the main text, the Lorentzian linewidth reduction is also a function of the Lorentzian linewidth of the pump. Here, we demonstrate this phenomenon experimentally. We use a different pump laser (Toptica, CTL 1550) whose Lorentzian linewidth decreases inversely proportional to pump power (see Supplementary Fig. 3a) [6].

Supplementary Fig. 3b presents the Lorentzian linewidth distribution of a soliton microcomb when the microresonator is pumped with a laser featuring different Lorentzian linewidths. As the pump linewidth decreases, the position of the quiet mode shifts towards the pump location. As plotted in Supplementary Fig. 3c, the Lorentzian linewidth reduction attained at the quiet mode increases linearly with the value of the Lorentzian linewidth of the pump. These trends are in agreement with the predictions set forth by equation (9) in the main text.

Supplementary Note 3: Frequency noise power spectral density and Lorentzian linewidth of the comb lines

The Lorentzian linewidth of a laser is usually obtained from its frequency noise power spectral density (PSD) $S_{\Delta\nu}(f)$. Here the frequency noise PSD for some representative comb lines are shown in Supplementary Fig. 4, which corresponds to the experimental results presented in Fig. 3 in the main text.

The Lorentzian linewidths of the comb lines are obtained based on the average value of the white-noise plateau within the 3-5 MHz region of $S_{\Delta\nu}(f)$, marked as the red lines in Supplementary Fig. 4. This region is chosen based on two considerations. Firstly, as can be seen from Supplementary Fig. 4a, the flicker noise caused by thermorefractive noise and pump technical noise dominates at low frequencies. Secondly, at high frequencies, the measured $S_{\Delta\nu}(f)$ is dominated by the white phase noise, which originates from the optical amplifiers ASE noise and the thermal noise of the measurement system [7]. The white phase noise corresponds to f^2 frequency noise, as shown in Supplementary Fig. 4b. To verify that the measured values are much higher than the measurement floor of our system, we measured the frequency noise PSD of other low linewidth lasers, which are shown in Supplementary Fig. 4b. It is shown that our measurement system can capture the frequency noise PSD $S_{\Delta\nu}(f)$ accurately up to 5 MHz.

Besides the white-noise plateau, one can observe frequency noise spikes in the PSD of most of the comb lines, which originate from the pump laser. Similar to the experiment presented in the main text, the magnitude of the those spikes changes with line number and gets suppressed at the fixed point, see Supplementary Fig. 4c.

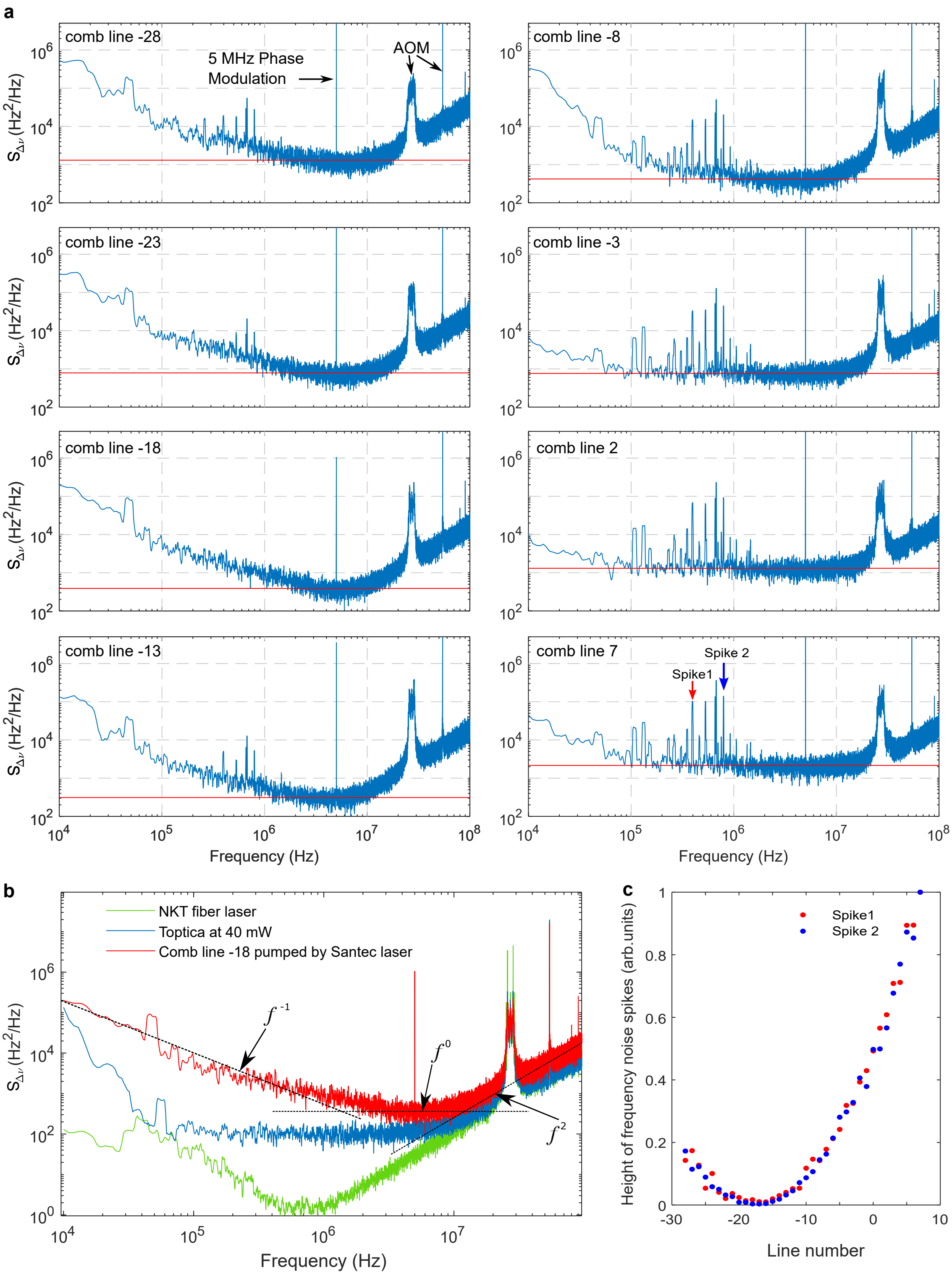

Supplementary Figure 4: **a** Frequency-noise power spectral density (PSD) $S_{\Delta\nu}$ of selected microcomb lines. The Lorentzian linewidth of comb lines are calculated based on the mean value of white-noise f^0 plateau in the PSD, as marked with the red lines. **b** Measured frequency-noise PSD of three lasers for comparison. **c** The relative height of two frequency noise spikes originating from pump laser. Spikes 1 and 2 are indicated in **a** (comb line 7).

Supplementary Note 4: Pump at another wavelength

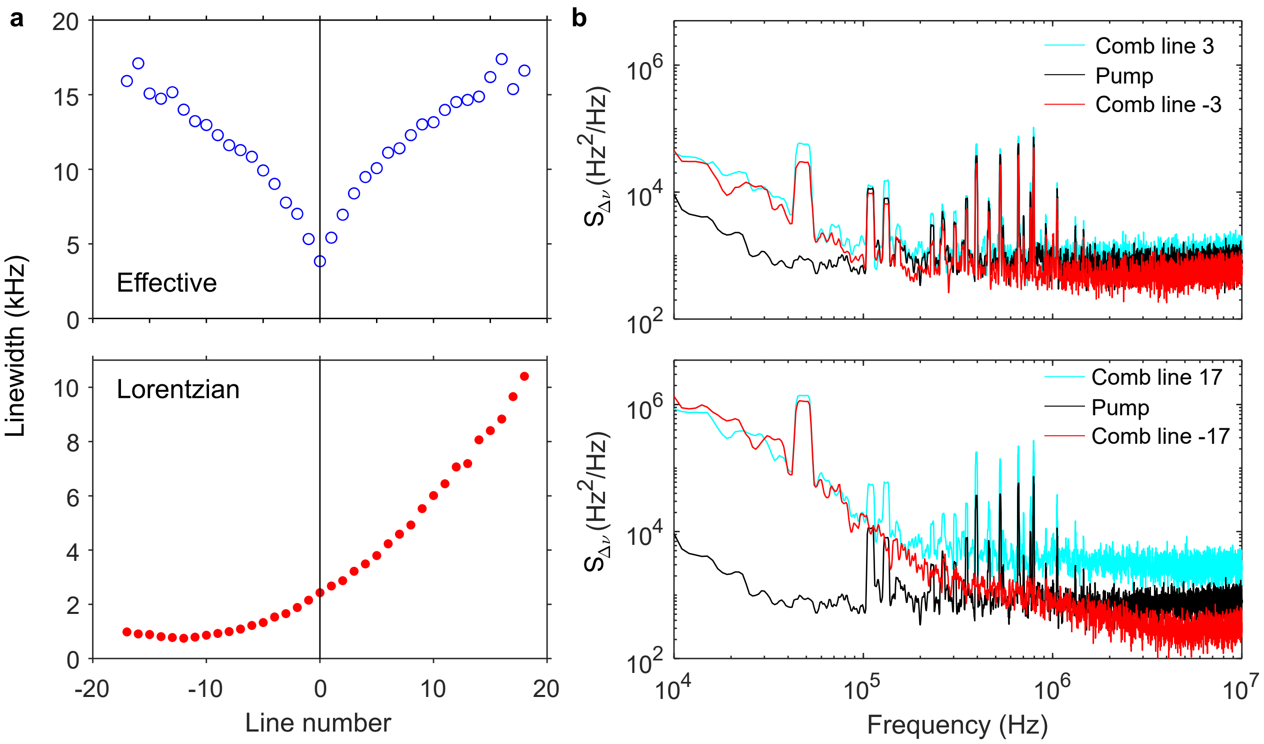

Supplementary Figure 5: **a** The effective linewidth and the Lorentzian linewidth when the pump (0 line number) is located at 1561 nm instead. **b** Frequency noise PSD for two pairs of comb lines symmetrically located close (top panel) and far (bottom panel) from the pump.

To perform a better comparison of the Lorentzian linewidth and effective linewidth, we provide further experimental results by pumping a different mode close to 1561 nm, i.e. close to the center bandwidth of our measurement system. The measured effective linewidth and Lorentzian linewidth of the comb lines are shown in Supplementary Fig. 5a.

The symmetric distribution of the effective linewidth around the pump can be understood from the frequency noise PSD, see Supplementary Fig. 5b. Because the effective linewidth is dominated by the low-frequency region in the PSD, it remains nearly the same for two comb lines with the same frequency distance from the pump, e,g., comb lines -17 and 17.

Supplementary References

1. V. Brasch, M. Geiselmann, T. Herr, G. Lihachev, M.H. Pfeiffer, M.L. Gorodetsky, T.J. Kippenberg. Science **351** 357-360 (2016)
2. A.B. Matsko, W. Liang, A.A. Savchenkov, D. Eliyahu and L. Maleki. Opt. Lett. **41** 2907-2910 (2016)
3. Q.F. Yang, X. Yi, K.Y. Yang and K. Vahala, Optica, **3**, 1132-1135 (2016).
4. X. Yi, Q.F. Yang, X. Zhang, K.Y. Yang, X. Li and K. Vahala, Nat. Commun. **8**, 14869 (2017).
5. Ó. B. Helgason, F.R. Arteaga-Sierra, Z. Ye, K. Twayana, P. A. Andrekson, M. Karlsson, J. Schröder, and V. Torres-Company. Nature Photonics **15** 305-310 (2021).
6. K. Petermann, *Laser diode modulation and noise*, Vol.3 (Springer Science & Business Media, 1991).
7. N. R. Newbury and W. C. Swann, J. Opt. Soc. Am. B **24**, 1756 (2007).
